# Investigating the Catalytic Influence of Boron on Ni-Co/Ca Catalysts for Improved Syngas Generation from Rice Straw Pyrolysis

**DOI:** 10.3390/molecules29081730

**Published:** 2024-04-11

**Authors:** Jiaxiang Wang, Luqi Wang, Yueyao Li

**Affiliations:** 1 School of Chemical and Environmental Engineering, Wuhan Polytechnic University, Wuhan 430023, China; wangjiaxiang370@163.com; 2 Anhui Agricultural University, Hefei 230036, China; 3 Institute of Tibet Plateau Ecology, Tibet Agricultural & Animal Husbandry University, Nyingchi 860000, China

**Keywords:** syngas production, biomass pyrolysis, B addition, Ni-Co/Ca catalysts, resource utilization

## Abstract

A series of boron-promoted Ni-Co/Ca catalysts were synthesized by the sol–gel method to enhance syngas generation from biomass pyrolysis. The efficiency of these catalysts was evaluated during the pyrolysis of rice straw in a fixed-bed reactor, varying the Ni/Co ratio, boron addition, calcination temperature, and residence time. The catalysts underwent comprehensive characterization using X-ray diffraction (XRD), Brunauer–Emmett–Teller (BET) analysis, scanning electron microscopy (SEM), and hydrogen temperature-programmed reduction (H_2_-TPR). The results indicated that the Ni-Co/Ca catalysts yielded superior syngas compared to singular Ni or Co catalysts, suggesting a synergistic interplay between nickel and cobalt. The incorporation of 4% boron significantly decreased the particle size of the active metals, enhancing both the catalytic activity and stability. Optimal syngas production was achieved under the following conditions: a biomass-to-catalyst mass ratio of 2:1, a Ni-Co ratio of 1:1, a calcination temperature of 400 °C, a pyrolysis temperature of 800 °C, and a 20 min residence time. These conditions led to a syngas yield of 431.8 mL/g, a 131.28% increase over the non-catalytic pyrolysis yield of 188.6 mL/g. This study not only demonstrates the potential of Ni-Co/Ca catalysts in biomass pyrolysis for syngas production but also provides a foundation for future catalyst performance optimization.

## 1. Introduction

As the world’s energy demand increases, fossil energy consumption generates a large amount of CO_2_. This situation can lead to serious energy security and ecological problems. On the one hand, the conversion of organic matter into fossil fuels takes millions of years, leading to the depletion of nonrenewable energy resources [1,2]. Furthermore, the combustion of fossil fuels releases sulfur dioxide (SO_2_), a toxic gas, and carbon dioxide (CO_2_), a potent greenhouse gas. These emissions contribute to environmental challenges such as acid rain and disrupt the ecological equilibrium [3]. I t has become a significant trend to meet the current energy demand while maintaining sustainability. In contrast to traditional fossil fuels, hydrogen (H_2_) is a clean energy source that produces only water and heat upon combustion. It does not emit greenhouse gases such as CO_2_, which contribute to pollution and global warming. Furthermore, hydrogen can be produced from renewable energy sources, reducing our dependence on nonrenewable fossil fuels [4]. This makes it a promising alternative for a more sustainable future. Lignocellulosic biomass is a promising renewable resource that is widely available and abundant in locations with large-scale planting, such as China. According to the National Report on the Comprehensive Utilization of Crop Straw by Chinese Government, in 2021, there were 647 million tons of crop straw utilized nationwide, indicating a wide range of utilization prospects for straw in China [5]. It has zero net CO_2_ emissions and is one of the most promising solutions to global warming caused by the transitional use of fossil resources. According to the International Energy Agency (IEA) report, the share of renewable energy in global electricity generation rose from 27% in 2019 to 29% in 2020. The use of bioenergy in industry increased by 3%. It is expected that in 2021, the proportion of renewable energy in electricity generation will reach a historical high of 30%. Consequently, biomass is expected to become a viable alternative to fossil energy [6,7,8].

Renewable energy can be transformed into a hydrogen-rich gas through two primary methods: thermochemical conversion and bioconversion. Thermochemical conversions stand out for their cost-effectiveness and the relative maturity of the technology. They excel in decomposing carbonaceous materials, breaking chemical bonds, and harnessing energy efficiently [9,10]. Pyrolysis involves transforming biomass into char, bio-oil, and gases—predominantly carbon monoxide (CO), hydrogen (H_2_), carbon dioxide (CO_2_), and methane (CH_4_)—within a high-temperature, oxygen-free environment. However, the process generates substantial quantities of bio-oil and CO_2_, making it increasingly critical to address the challenge of effectively removing these by-products [11].

When comparing biomass energy conversion technologies such as pyrolysis and gasification, although gasification can directly convert biomass into syngas at higher temperatures, expanding its range of chemical product applications, this technology faces challenges including higher energy consumption, strict feedstock pretreatment requirements, higher initial investment and operational costs, and the potential production of difficult-to-handle by-products, limiting its widespread application. In contrast, pyrolysis technology, with its lower operational temperatures, strong adaptability to feedstocks, relatively lower technical and economic barriers, and the environmental and economic benefits of its by-product, biochar, offers a more efficient, flexible, environmentally friendly, and economically viable pathway for biomass energy conversion. Pyrolysis not only effectively reduces the complexity and cost of by-product handling but also enhances the yield and quality of syngas through optimized operational conditions, achieving the high-value utilization of biomass resources [12]. Therefore, based on a comprehensive assessment of energy conversion efficiency, technological adaptability, environmental benefits, and economic considerations, pyrolysis emerges as the preferred technology pathway for syngas production, aiming to meet production needs while delivering additional environmental and economic benefits.

In generating hydrogen-rich synthesis gas (syngas) through biomass pyrolysis, the incorporation of catalysts plays a pivotal role not only in amplifying the gas yield but also in enhancing the selectivity towards the desired product [13,14]. The selection of a suitable catalyst can promote tar reforming and selectively control the pyrolysis products through CO_2_ adsorption [15]. Nickel-based catalysts are recognized for their effectiveness in facilitating hydrogenation reactions. They offer several advantages, including high catalytic activity, cost-effectiveness, and wide availability. Given their exceptional performance in catalysis, they are broadly utilized in the steam reforming of biomass to generate hydrogen [16]. Additionally, cobalt (Co) plays a crucial role in facilitating the cleavage of C-C bonds and amplifying water–gas shift reactions, thereby promoting H_2_ generation [17,18]. Furthermore, calcium oxide (CaO) is abundant and inexpensive, reacting easily with CO_2_ at 650 °C to 750 °C for CO_2_ absorption. At 850 °C to 900 °C, the generated calcium carbonate (CaCO_3_) is reduced for recycling [19].

Xu et al. [20] developed a series of Ni/CaO catalysts and systematically analyzed their efficacy in the catalytic pyrolysis of herbal residues. The results demonstrated that incorporating Ni into CaO could effectively enhance its anti-deactivation capability for the cyclic adsorption of CO_2_. Moreover, at 700 °C, the addition of a 10% Ni/CaO catalyst not only improved the gas yield to 325.3 mL/g but also decreased the tar output. Lu et al. [21] investigated the Ni/Ca-promoted Fe catalyst’s potential for sustainable hydrogen generation, revealing that the incorporation of Ca and Ni effectively promoted the dispersion of Fe particles. The Fe/Ca/Ni catalyst system significantly increased the production of light oil and hydrogen-rich gas. For instance, San-José-Alonso et al. [22] documented that the 5Fe1.5Ca0.8Ni catalyst reached a maximum hydrogen yield of 91.19 mL/g at 750 °C. The investigation into alumina-supported NiCo catalysts aimed at identifying active and stable catalysts. The findings indicated that catalysts containing both Co and NiCo facilitated significant carbon build-up during the dry reforming of methane, which subsequently led to diminished catalytic activity. In contrast, Santamaria et al. [23] reported that catalysts with a higher Co content exhibited enhanced catalytic performance, and they conducted a similar study and found that the formation of Ni-Co alloys in bimetallic catalysts prevented the formation of Co^0^ and thus improved the catalyst activity. Additionally, Wu et al. [24] discovered that adding boron facilitated the catalytic performance and selectivity of the NiCeZr catalyst in ethanol steam reforming. It enhanced the decomposition of acetaldehyde and reduced the selectivity for acetone. This reduced the formation of coke. Singh et al. [25] demonstrated that adding 1% and 2% boron to Ni/SBA-15 could enhance the conversion of CH_4_ and CO_2_ in the dry reforming of ethanol while effectively controlling the particle size of NiO. However, excessive boron may obstruct the pore channels of SBA-15 and consequently diminish catalytic activity. Thus, it is imperative to select an optimal amount of boron doping.

Building on the findings of the previous studies, we employed the sol–gel method to synthesize a series of Ni-Co/Ca catalysts, aiming to preserve their porous structure and thermal stability [26]. Our study investigated the effects of incorporating boron into Ni-Co catalysts to improve their catalytic activity in producing syngas. Additionally, we evaluated how variations in active metal content, calcination temperature, residence time, and pyrolysis temperature affect the syngas yield from biomass pyrolysis, aiming to experimentally determine the most favorable conditions. Furthermore, we examined the effect of boron addition on catalyst activity and employed characterization techniques, including XRD, H_2_-TPR, BET, and SEM, to assess differences in the physicochemical structures of the catalysts.

## 2. Results

### 2.1. Characterizations of Catalysts

#### 2.1.1. XRD

**Figure 1:**
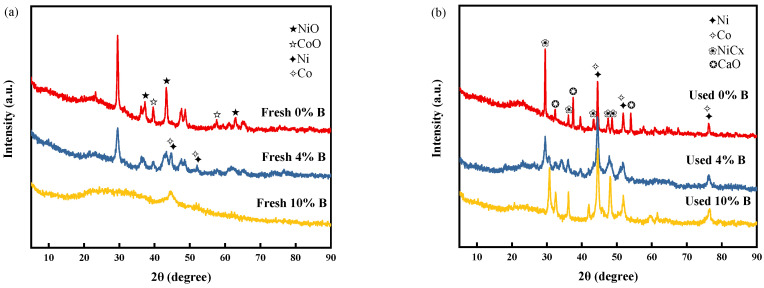
XRD pattern of the fresh and used catalysts: (**a**) fresh catalysts, (**b**) used catalysts.

Figure 1 displays (XRD) patterns for both fresh and utilized catalysts. In the case of the fresh catalyst, prior to the incorporation of boron, distinct peaks characteristic of NiO were identified at 2θ angles of 37.25°, 43.29°, and 62.85° (JCPDS No. 44-1159). Similarly, peaks denoting the presence of CoO were discerned at 2θ = 36.51°, 42.41°, and 61.52°, as per JCPDS No. 43-1004. These observations confirm that the active metals were initially present in their oxide forms. Upon introducing boron into the catalyst composition, a gradual attenuation in the intensity of these oxide peaks was observed. This trend persisted until the addition of 10% boron, beyond which the original oxide peaks vanished, leaving behind only faint characteristic peaks of Ni and Co. This phenomenon suggested that boron addition effectively enhances the dispersion of active metals within the catalyst matrix [27]. The particle size of NiO in the fresh catalyst, before the addition of boron, was determined to be 21.4 nm using Scherrer’s formula. When the boron content was increased to 4%, a notable reduction in the NiO particle size to 5.0 nm was observed. This significant decrease in particle size further corroborated the role of boron in diminishing the particle size of the catalysts. Such findings are consistent with the results presented by Fouskas et al. [28]. In the used catalyst samples, those devoid of boron supplementation exhibited distinctive peaks corresponding to CaO. Conversely, such characteristic peaks appeared subdued in the boron-enriched samples, suggesting that boron’s inclusion may deter the sintering of CaO under elevated temperatures. It is noteworthy that no characteristic peaks of CaCO_3_ were found in the XRD pattern, which may be due to the presence of H_2_ in the generated syngas, which reacts with CaCO_3_ to form CaO [29]. Furthermore, all utilized catalysts presented characteristic peaks indicative of NiCx, attributable to carbon deposition [30]. However, the incorporation of boron was observed to diminish the intensity of these peaks, indicating boron’s efficacy in mitigating carbon deposition [31]. A comparative analysis of the catalysts, each incorporating boron at concentrations of 4% and 10%, unveiled a discernible contrast in their peak intensities. Specifically, the catalyst with a 4% boron content exhibited a diminished peak intensity relative to its 10% boron counterpart. This observation underscores the pivotal role of boron content in enhancing the crystallinity and mitigating carbon deposition on the catalysts. Furthermore, a universal increase in particle size was observed across all utilized catalysts when compared to their pristine counterparts (see Table 1 below), signifying that metal aggregation transpired to a certain extent during the catalytic reaction process.

#### 2.1.2. N_2_ Adsorption/Desorption

**Figure 2:**
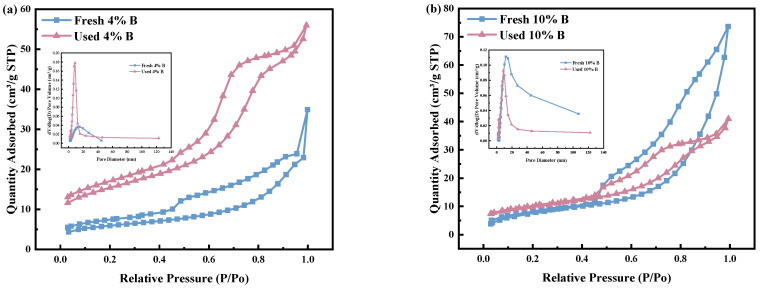
N_2_ adsorption/desorption isotherms and pore distributions of fresh and used catalysts: (**a**) 4% B, (**b**) 10% B.

**Figure 3:**
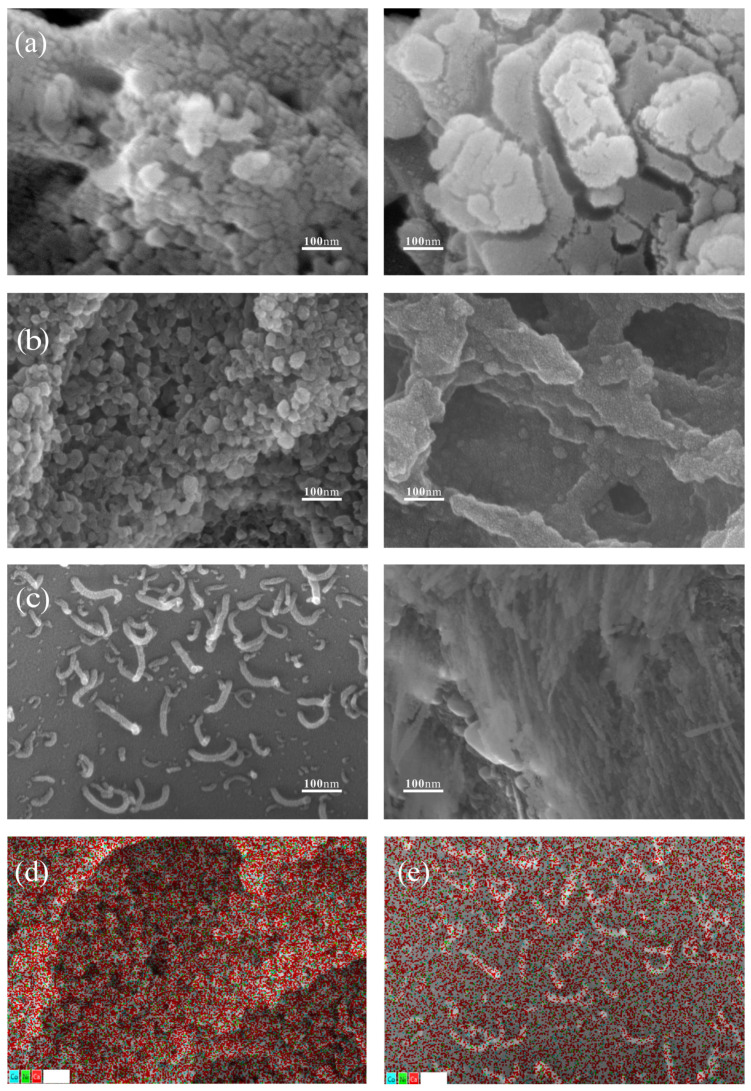
SEM results of fresh (**left**) and used (**right**) catalysts. (**a**) 0% B; (**b**) 4% B; (**c**) 10% B; (**d**) fresh 4% B EDS; (**e**) fresh 10% B EDS.

The physicochemical attributes of both fresh and spent catalysts are delineated in Table 2. The nitrogen adsorption–desorption isotherms, along with the pore size distribution for these catalysts, are depicted in Figure 2. The specific surface areas of the fresh catalysts were observed to be in the range of 20–30 m^2^/g, which notably tended to be augmented subsequent to utilization. This increase was particularly pronounced for catalysts with a 4% boron composition, a phenomenon attributable to the redistribution of catalyst particles post use and the emergence of a more porous network structure (Figure 3) [32]. Such structural modifications not only enhanced the pore volume but also augmented the available pore space conducive to the incorporation of active metal species, thereby elevating the specific surface area of the catalyst. Conversely, catalysts composed of 10% boron exhibited a reduction in pore volume. This phenomenon was linked to the conversion of numerous rod-like structures into more voluminous, filamentous configurations post use, which, while occupying substantial pore channels, also enriched the catalyst’s specific surface area. This structural evolution was crucial as it significantly influenced the catalyst’s functionality and durability. The augmented specific surface area played a pivotal role in enhancing catalytic activity; however, the diminished pore volume could have potentially impinged on the pore architecture and the catalyst’s long-term stability.

Moreover, in both pristine and spent catalysts, the inclusion of 10% boron resulted in larger pore sizes compared to those containing 4% boron. This observation underscored the role of boron in augmenting the pore dimensions [33]. Table 2 reveals that the fresh catalyst enriched with 10% boron exhibited a greater pore volume compared to its used counterpart, a contrast not observed in the catalyst with 4% boron addition. According to the IUPAC classification, all catalysts displayed characteristic type IV isotherms, with fresh catalysts showing H3 hysteresis loops and used catalysts exhibiting H4 hysteresis loops. This indicated that the fresh catalysts possessed mesoporous structures with slit-like pores created by loosely aggregated particles, whereas the used catalysts maintained the presence of slit pores [34,35]. The data presented in Table 2, alongside the pore size distribution illustrated in Figure 2, reveal the existence of mesoporous structures within the catalysts, characterized by pore sizes predominantly ranging from 0 to 20 nm. Notably, the pore size distribution underwent a modification upon the catalysts’ utilization, evidenced by a reduction in pore sizes to varying extents. Specifically, Table 2 demonstrates that the catalyst containing 10% boron experienced a decrease in pore size by 0.9 nm following its application, whereas the catalyst comprising 4% boron witnessed a reduction of approximately 0.6 nm in pore size. This observation underscored the dynamic nature of the catalysts’ mesostructures under operational conditions and highlighted the influence of boron content on the resilience of pore architecture post utilization.

#### 2.1.3. SEM

SEM analysis was used to investigate the surface morphology of the catalysts. Figure 3 shows the SEM images of the fresh catalyst (left) and used catalyst (right) and their EDS images. The surface of the fresh catalyst without B addition exhibited a bumpy structure (Figure 3a left), with surface masses increasing significantly after use (Figure 3a right). In contrast, all fresh catalysts with B addition displayed specific structures; for example, the surface of the fresh catalyst with 4% B addition (Figure 3b left) showed a tight accumulation of spherical particles [36], which was associated with NiO or/and CoO particles [15]. This structure effectively promoted gas diffusion within the catalyst, thus enhancing catalytic performance. In contrast, the fresh catalyst with 10% B addition (Figure 3c left) had a smoother carrier surface with numerous strip-like columnar structures. Additionally, the morphology of these B-loaded catalysts, when used, differed significantly from those without B. The surface of the catalysts without B was disordered and accompanied by lumps. The catalysts loaded with 4% B exhibited a porous mesh structure, increasing the specific surface area, whereas those loaded with 10% B developed a filamentous structure after use. Moreover, the analysis of EDS images for the freshly prepared 4% B-enhanced Ni-Co/Ca catalyst (Figure 3d) and the 10% B-enhanced Ni-Co/Ca catalyst (Figure 3e) not only confirmed the successful loading of active metal elements onto the surface of the catalysts but also unveiled the uniform dispersion of these metallic elements across the catalyst surfaces.

#### 2.1.4. H_2_-TPR

**Figure 4:**
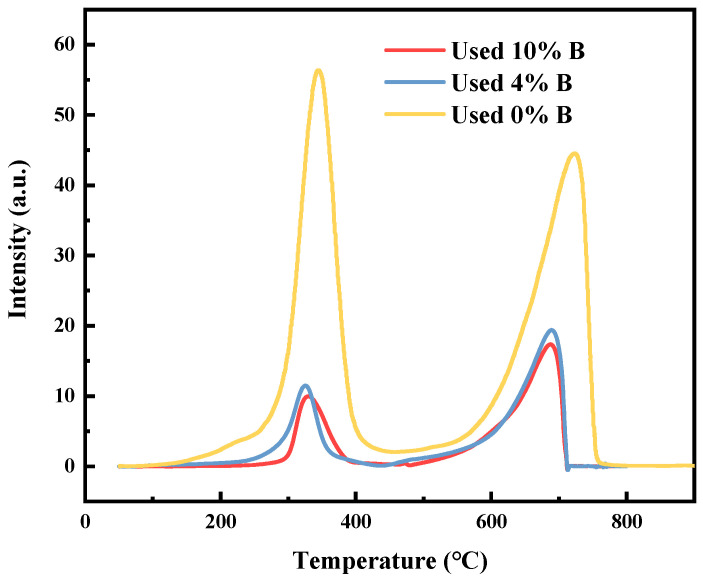
H_2_-TPR profiles of catalysts.

To delve deeper into the stability of the catalyst, especially under actual use conditions, H_2_-TPR measurements were conducted on the used catalysts, as shown in Figure 4. This analysis aimed to examine the reduction behavior of the catalysts after use and the interaction between nickel species and the carrier, thereby offering direct evidence of catalyst stability. Within the temperature range of 200 to 700 °C, two significant H_2_ consumption peaks were observed, corresponding to the reduction of exposed oxide species and their interaction with the carrier [37].

Specifically, the reduction peak near 310 °C primarily originated from the reduction of free oxide species on the carrier surface, while the significant reduction peak near 690 °C was due to the reduction of oxide species that strongly interacted with the carrier [38], a process that consumed a substantial amount of H_2_ [39]. The presence and characteristics of these reduction peaks indicated that the catalysts retained their reduction properties even after use, serving as an important sign of catalyst stability.

Comparing the H_2_-TPR analysis of the three catalysts, we found that the reduction temperatures of these catalysts were essentially the same, indicating that the role of the metal carriers within the catalysts did not change significantly before and after the addition of boron, further emphasizing the stability of the catalysts during the reaction process. Notably, the catalysts without the addition of boron exhibited stronger reduction peaks, suggesting that a larger particle size of NiO/CoO facilitated the reduction of oxides. This not only revealed the impact of boron addition on the catalyst particle size but also highlighted the potential role of boron in enhancing catalyst resistance to carbon build-up and promoting catalyst stability [40]. Conducting H_2_-TPR tests on used catalysts revealed their retained reduction properties after reaction, providing important evidence of the catalyst’s ability to resist carbon accumulation and maintain activity.

### 2.2. Analysis of Influencing Factors

#### 2.2.1. Ni/Co Ratio

**Figure 5:**
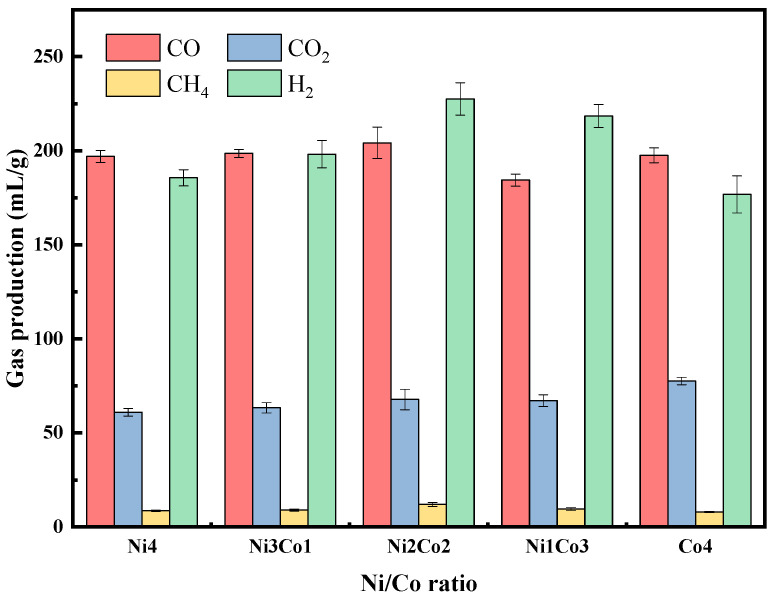
Effect of Ni-to-Co ratio on pyrolysis gas composition with 4% B catalysts.

Figure 5 shows the effect of the Ni and Co active metal ratios on the pyrolysis gas yield. The four main pyrolysis gas products from rice straw are H_2_, CO, CO_2_, and CH_4_. It can be observed that single Ni catalysts or Co catalysts are much less effective than Ni Co bimetallic catalysts. Due to the synergy, the H_2_ yield with Ni-Co bimetallic catalysts is around 200 mL/g. Li et al. [39] demonstrated that bimetallic Ni-Co catalysts had a slightly higher conversion rate of CH_4_ and CO_2_ in methane reforming with CO_2_ (MRC) compared to monometallic Ni or Co catalysts, attributed to the synergistic effect of the Ni and Co transition metals. However, the highest H_2_ yield of 227.6 mL/g was obtained at a Ni-to-Co ratio of 1:1. In addition, the yield of CO was relatively stable, and the yield reached its peak when the ratio of Ni-Co was 1: 1, which was 204.2 mL/g, indicating the similar catalytic performance in generating CO. With the continuous addition of Co, the CO_2_ content increased gradually, reaching its maximum when only Co was used. This shows that CO and CO_2_ are more likely to be produced in the presence of Co.

#### 2.2.2. Residence Time

**Figure 6:**
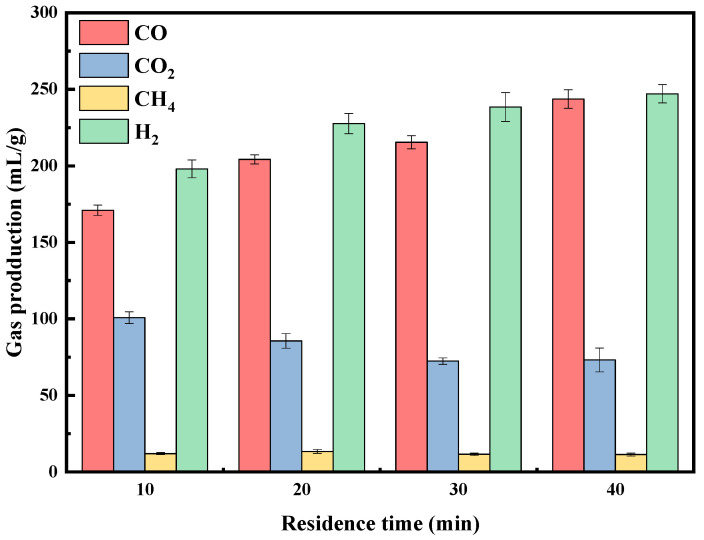
Effect of residence time on pyrolysis gas composition with 4% B catalysts.

Residence time refers to the duration that the catalyst remains in contact with the reactants under specific conditions. A longer residence time favors secondary pyrolysis, reducing the yield of bio-oil and increasing the production of gaseous components [41]. The pyrolysis experiment on rice straw was conducted at 800 °C [42] using a 4% B-Ni-Co/Ca catalyst with a Ni-to-Co ratio of 1:1. Figure 6 shows the effect of residence time on the distribution of gas products. H_2_ and CO accounted for the majority of the generated gas components, accounting for more than 75% of the total gas. Notably, after 20 min, including at the 20 min mark, the total syngas (CO + H_2_) content exceeded 80%. However, Lan et al. [43] reported the highest syngas level at only 78.9%. With increasing residence time, the total gas volume rose from 481.8 mL/g at 10 min to 565.2 mL/g at 40 min. The variation in the production of H_2_ and CH_4_ was not significant. However, the CO_2_ content decreased from 100.8 mL/g to 73.2 mL/g, and the CO content gradually increased from 171.0 mL/g to 243.6 mL/g, mainly due to the conversion of CO_2_ to CO and the further cracking of tar (C + CO_2_ → 2CO, CO_2_ + H_2_ → CO + H_2_O and CnHm + nCO_2_ → 2nCO + m/2H_2_) [44,45,46]. In summary, a longer residence time leads to more complete straw pyrolysis and a higher production of syngas.

#### 2.2.3. Calcination Temperature

**Figure 7:**
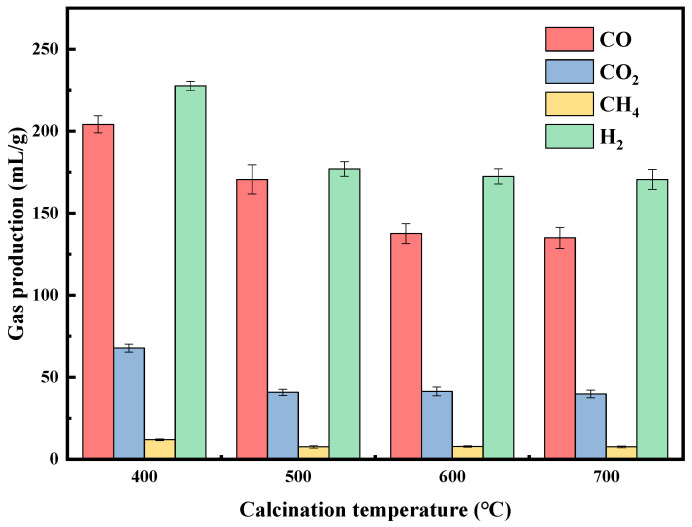
Effect of calcination temperature on pyrolysis gas composition with 4% B catalysts.

Changes in calcination temperature significantly impact the activity and stability of catalysts during the catalytic process [47]. As shown in Figure 7, as the calcination temperature increased, the total gas emission decreased [42]. CO, H_2_, and CO_2_ all decreased significantly when the calcination temperature was increased from 400 to 500 °C. Emissions continued to decrease beyond 500 °C though by a lesser amount. This decrease may be attributed to the higher calcination temperature, accelerating CaO sintering. Wang et al. [48] also found that an increase in calcination temperature leads to the sintering and aggregation of metal oxide particles. Furthermore, Ma et al. [49] determined that excessively high calcination temperatures significantly decrease the surface area and pore volume of the catalyst while simultaneously forming metal clusters, leading to a deterioration in catalyst performance. Additionally, catalysts calcined at low temperatures may exhibit lower interaction between the active component and the carrier due to minimal carrier sintering, resulting in the highest syngas yield of 436.2 mL/g (H_2_: 227.6 mL/g, CO: 208.6 mL/g) at 400 °C with the 4% B-Ni-Co/Ca catalyst.

#### 2.2.4. Addition of B

**Figure 8:**
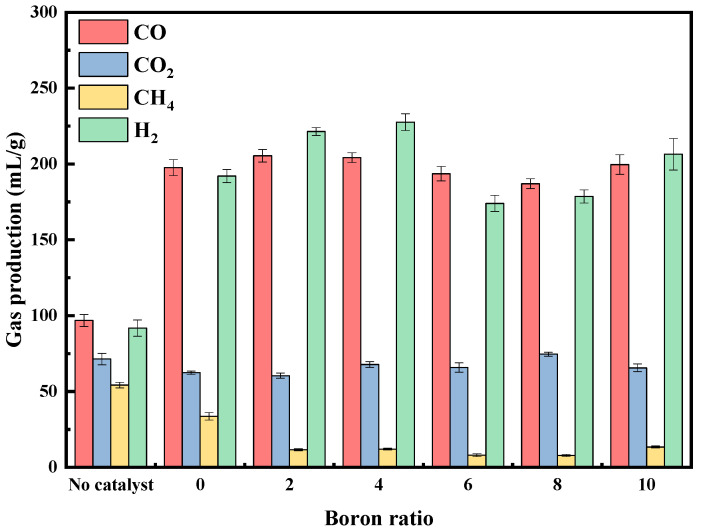
Effect of different boron additions on gas yield.

Due to its unique properties, boron is considered an important component in non-metallic catalysts [50]. Therefore, the study explored the effect of boron addition (0–10%) on gas content changes at calcination temperatures of 400 °C and pyrolysis temperatures of 800 °C. The results were compared with the gas production without a catalyst, as shown in Figure 8. It was evident that the addition of boron exhibited an interesting trend in terms of overall gas production. It displayed a pattern of increment followed by decrement and then increment again with respect to both H_2_ and CO production. The catalyst activity exhibited a gradual increase, and the syngas concentration progressively rose when boron was added from none to 4%. Among them, the syngas concentration reached its peak at 436.2 mL/g with the 4% addition. Compared to 384.6 mL/g without boron, the increase was 13.42%. When compared to 188.6 mL/g without a catalyst, the increase was 131.28%. At higher boron additions (6% and 8%), the catalytic activity of the catalysts was suboptimal, and the total syngas production was lower than that of the catalysts without boron addition. Furthermore, the yields of H_2_ and CO were comparable in the absence of catalyst presence. Even when the boron-free catalyst was added, the catalysts exhibited similar enhancements of CO and H_2_ yields. The yield of CH_4_ was significantly reduced by the addition of boron to the catalyst. However, the amount of boron had minimal effect on the yield of CH_4_. Furthermore, the XRD and SEM results revealed that boron addition reduced metal particle size and improved metal distribution on the catalyst surface, forming a more ordered structure. This enhanced the catalytic performance of the catalyst.

**Figure 9:**
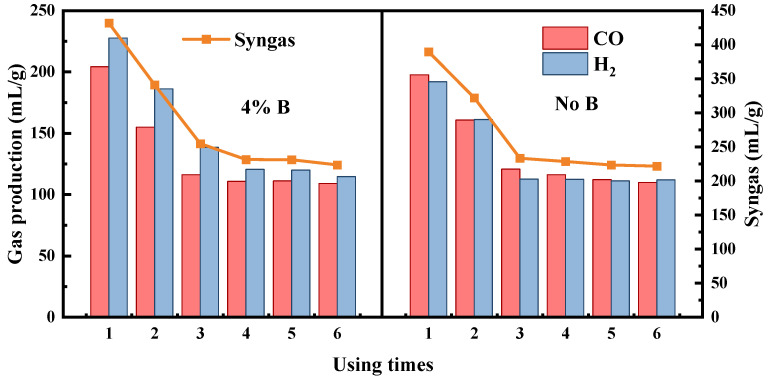
Lifetime testing of different catalysts.

The catalyst with 4% boron addition demonstrated evidently enhanced catalytic performance. Its longevity, as depicted in Figure 9, was evaluated and compared to that of the catalyst without boron. In the initial three trials, the catalyst enriched with 4% boron markedly increased the syngas yield, producing a higher proportion of H_2_ relative to CO. Conversely, with the boron-free catalyst, the yields of H_2_ and CO were nearly identical. After adding boron, fluctuations in the H_2_ levels significantly surpassed those in CO, suggesting that boron primarily affected H_2_ production. This phenomenon could be attributed to boron’s role in advancing the carbonation reaction while exerting minimal impact on CO production [51]. The activity of the 4% boron-containing catalyst significantly diminished after four uses, with the syngas yield dropping to merely 223.6 mL/g. Conversely, the boron-free catalyst almost lost its efficacy after three uses, with a syngas yield of 221.8 mL/g. Nevertheless, these yields were superior to those obtained without any catalyst, which were recorded at 188.6 mL/g.

Further investigations, using XRD characterization, revealed the presence of NiCx, implicating carbon accumulation on the surface as a key factor in catalyst deactivation. The addition of boron was observed to promote an orderly distribution of metal on the carrier surface, enhancing the catalyst’s resistance to deactivation. This is in line with findings by Fouskas et al. [28], demonstrating that boron addition not only reduces the particle size of Ni but also impedes catalyst coking, consequently extending the operational lifespan of the catalyst.

## 3. Materials and Methods

### 3.1. Catalyst Preparation

The biomass used in the experiments was rice straw, which was sourced from Wuhan, Hubei Province. The main characteristics of rice straw were shown in our previous studies and are presented in Table 3 [52]. The rice straw was pulverized and sifted to produce a powder with particle sizes ranging from 1 to 2 mm, suitable for experimental use. The Ni(NO_3_)_2_·6H_2_O, Ca(NO_3_)_2_·4H_2_O, Co(NO_3_)_2_·6H_2_O, C_6_H_8_O_7_·H_2_O, and (CH_2_OH)_2_ required for the experiments were purchased from Sinopharm Chemical Reagent Co, Ltd. And, the boric acid was purchased from Damao Chemical Reagent Factory. The purchased reagents are of analytical grade and can be used without any further processing.

A series of B-modified Ni-Co catalysts was prepared using the sol–gel method. In a 100 mL beaker, 6.60 g of Ca(NO_3_)_2_·4H_2_O was dissolved, followed by the addition of 1.98 g of Ni(NO_3_)_2_·6H_2_O and 1.98 g of Co(NO_3_)_2_·6H_2_O, with stirring for 30 min. To the mixed solution, 10.29 g of C_6_H_8_O_7_·H_2_O and 5.46 mL of (CH_2_OH)_2_ were added, and the mixture was stirred for an additional 30 min. The mixed solution was then transferred to an oil bath and stirred continuously at 95 °C for several hours until a gel-like solution formed. The solution was subsequently dried in an oven at 110 °C for 12 h to obtain the Ni-Co/Ca catalyst. Next, 0.46 g of boric acid was added to the catalyst, dissolved in the appropriate amount of deionized water. The solution was then placed in an oil bath at 95 °C for 2 h, afterwards removed and dried in an oven at 110 °C for 12 h. Subsequently, it was subjected to an inert atmosphere (N_2_) and calcined at 400 °C for 2 h to obtain a 4% B-Ni-Co/Ca catalyst. By adjusting the quantity of boric acid introduced and using a consistent methodology, catalysts with varying boron content ranging from 0% to 10% could be synthesized.

### 3.2. Catalyst Characterization

The crystal structure of the catalyst was examined using a Rigaku Smart Lab SE instrument with Cu-Kα radiation, scanning from 5° to 90° at a rate of 5°/min, under working conditions of 40 kV and 40 mA.

A ASAP 2460 instrument (Micromeritics, Norcross, GA, USA) was used to assess the specific surface area, pore volume, and average pore size of the catalysts. The samples were first degassed under vacuum at 120 °C for 6 h before the N_2_ adsorption–desorption isotherms were recorded at 77 K. The calculations for the specific surface area and the pore size distribution of the samples were performed using the BET and BJH methods, respectively.

The sample surface topography was determined in a ZEISS Gemini 300 instrument. Trace samples were directly applied to electrically conductive adhesive and then coated with gold at 10 mA using an SC7620 sputter coater (Oxford Quorum, East Sussex, UK). The specimen was subsequently imaged at 3 kV using a Gemini SEM300 (ZEISS, Oberkochen, Germany). At the time of spectrum mapping, the acceleration voltage was 15 kV, and the detector was an SE2 electron detector.

The programmed temperature rise reduction (TPR) technique was utilized to ascertain the reduction temperatures for various metal phases within the catalysts. These tests were conducted using a BELCAT II system (Microtrac, Osaka, Japan). For the pre-treatment process, each catalyst sample, weighing between 30 and 40 mg, was gradually heated from ambient temperature to 300 °C at a rate of 10 °C/min, followed by purging with He gas (50 mL/min) for 1 h. After cooling to 50 °C, the samples were exposed to a 10% H_2_/Ar mixture (50 mL/min) for 0.5 h, and then the temperature was increased to 800 °C at a rate of 10 °C/min for desorption. The presence of reducing gas was monitored using a thermal conductivity detector (TCD).

### 3.3. Catalytic Pyrolysis

The experiments were conducted in a BTF-1200C fixed-bed pyrolysis furnace, where the straw and catalyst were placed in separate quartz boats, with a mass ratio of 2 g:1 g [53]. Before the investigation began, N_2_ (100 mL/min) was introduced into the tube furnace for 30 min to create an inert environment and prevent biomass combustion. Subsequently, the furnace temperature was increased to the set temperature of 800 °C at a heating rate of 10 °C/min. After a specified reaction time, the gas generated in the tube furnace was expelled into a gas collection bag using N_2_. The collected gas was then analyzed using a Gasboard-3100 infrared gas analyzer, which determined the concentrations of CH_4_, H_2_, CO, and CO_2_.

## 4. Conclusions

A comprehensive array of Ni-Co/Ca catalysts was synthesized using the sol–gel method to evaluate the influence of various parameters on hydrogen production during rice straw pyrolysis. These parameters encompassed calcination temperatures, residence times, Ni-Co ratios, and boron (B) addition. Catalysts designated as 4% B-Ni-Co/Ca, characterized by their fluffy and porous morphology and smaller particle size, were synthesized under specific conditions, including a calcination temperature of 400 °C, a Ni-Co ratio of 1:1, and a 4% boron addition. These catalysts achieved an exceptional total syngas yield of 436.2 mL/g, comprising 227.6 mL/g of H_2_ and 208.6 mL/g of CO, at a pyrolysis temperature of 800 °C and a biomass-to-catalyst ratio of 2:1. The results highlighted a synergistic interaction between nickel and cobalt in facilitating biomass pyrolysis for syngas production. Furthermore, the addition of boron not only improved the distribution of active metals on the carrier surface but also contributed to reducing the size of active metal particles. During catalyst lifetime assessments, the inclusion of boron was found to retard catalyst deactivation, thereby exhibiting its anti-deactivation properties.

## Figures and Tables

**Table 1:** Particle size in the sample according to the Scherrer formula.

Samples	Fresh 0% B (nm)	Used 0% B (nm)	Fresh 4% B (nm)	Used 4% B (nm)	Fresh 10% B (nm)	Used 10% B (nm)
NiO	21.4	65.8	5.0	-	-	-
Ni	-	31.7	8.8	13.2	10.1	14.8
CoO	20.7	28.5	4.7	-	-	-
Co	-	36.9	6.8	19.4	11.2	16.0
CaO	-	66.4	-	-	-	-

**Table 2:** Textural properties of the fresh and deactivated catalysts.

Catalyst	S_BET_ (m^2^/g)	Pore Volume (cm^3^/g)	Pore Size (nm)
10% B	29.0	0.11	7.3
4% B	20.4	0.05	6.9
Used 10% B	33.8	0.06	6.4
Used 4% B	53.2	0.09	6.3

**Table 3:** Proximate and ultimate analyses of rice straw.

Sample	Ultimate Analysis (wt.%)					Proximate Analysis (wt.%)			
	C	H	O *	N	S	M	A	V	FC
Rice straw	39.67	5.73	39.51	0.84	0.16	4.21	14.09	76.33	5.37

* The oxygen (O) content was determined by difference. M: moisture content; V: volatile matters; A: ash; FC: fixed carbon.

## Data Availability

The data from this research are available upon request from the corresponding author. The data are not publicly available because they are part of the ongoing research of the authors.
